# Constitutive knockout of interleukin-6 ameliorates memory deficits and entorhinal astrocytosis in the MRL/lpr mouse model of neuropsychiatric lupus

**DOI:** 10.1186/s12974-024-03085-9

**Published:** 2024-04-10

**Authors:** Joshua Reynolds, Michelle Huang, Yaxi Li, Myriam Meineck, Tamara Moeckel, Julia Weinmann-Menke, Chandra Mohan, Andreas Schwarting, Chaim Putterman

**Affiliations:** 1 Albert Einstein College of Medicine, 1300 Morris Park Avenue, Bronx, New York, NY USA; 2 University of Houston, Houston, TX USA; 3 University Medical Center of the Johannes Gutenberg University, University of Mainz, Mainz, Germany; 4 Azrieli Faculty of Medicine, Bar-Ilan University, Zefat, Israel

**Keywords:** NPSLE, Interleukin-6, Astrocytosis, Entorhinal cortex, Memory deficits

## Abstract

**Background:**

Neuropsychiatric lupus (NPSLE) describes the cognitive, memory, and affective emotional burdens faced by many lupus patients. While NPSLE’s pathogenesis has not been fully elucidated, clinical imaging studies and cerebrospinal fluid (CSF) findings, namely elevated interleukin-6 (IL-6) levels, point to ongoing neuroinflammation in affected patients. Not only linked to systemic autoimmunity, IL-6 can also activate neurotoxic glial cells the brain. A prior pre-clinical study demonstrated that IL-6 can acutely induce a loss of sucrose preference; the present study sought to assess the necessity of chronic IL-6 exposure in the NPSLE-like disease of MRL/lpr lupus mice.

**Methods:**

We quantified 1308 proteins in individual serum or pooled CSF samples from MRL/lpr and control MRL/mpj mice using protein microarrays. Serum IL-6 levels were plotted against characteristic NPSLE neurobehavioral deficits. Next, IL-6 knockout MRL/lpr (IL-6 KO; n = 15) and IL-6 wildtype MRL/lpr mice (IL-6 WT; n = 15) underwent behavioral testing, focusing on murine correlates of learning and memory deficits, depression, and anxiety. Using qPCR, we quantified the expression of inflammatory genes in the cortex and hippocampus of MRL/lpr IL-6 KO and WT mice. Immunofluorescent staining was performed to quantify numbers of microglia (Iba1 +) and astrocytes (GFAP +) in multiple cortical regions, the hippocampus, and the amygdala.

**Results:**

MRL/lpr CSF analyses revealed increases in IL-17, MCP-1, TNF-α, and IL-6 (a priori p-value < 0.1). Serum levels of IL-6 correlated with learning and memory performance (R^2^ = 0.58; p = 0.03), but not motivated behavior, in MRL/lpr mice. Compared to MRL/lpr IL-6 WT, IL-6 KO mice exhibited improved novelty preference on object placement (45.4% vs 60.2%, p < 0.0001) and object recognition (48.9% vs 67.9%, p = 0.002) but equivalent performance in tests for anxiety-like disease and depression-like behavior. IL-6 KO mice displayed decreased cortical expression of *aif1* (microglia; p = 0.049) and *gfap* (astrocytes; p = 0.044). Correspondingly, IL-6 KO mice exhibited decreased density of GFAP + cells compared to IL-6 WT in the entorhinal cortex (89 vs 148 cells/mm^2^, p = 0.037), an area vital to memory.

**Conclusions:**

The inflammatory composition of MRL/lpr CSF resembles that of human NPSLE patients. Increased in the CNS, IL-6 is necessary to the development of learning and memory deficits in the MRL/lpr model of NPSLE. Furthermore, the stimulation of entorhinal astrocytosis appears to be a key mechanism by which IL-6 promotes these behavioral deficits.

**Supplementary Information:**

The online version contains supplementary material available at 10.1186/s12974-024-03085-9.

## Background

Among its many manifestations, systemic lupus erythematosus (SLE) impacts the central nervous system (CNS) in about 20–40% of lupus patients [1]. Occurring primarily in women between the second and fifth decades of their lives [2, 3], SLE’s neurologic sequelae can be associated with either focal pathologies relating to vascular disease or diffuse symptoms of unknown etiology. These diffuse neuropsychiatric features of lupus, collectively referred to as NPSLE, include cognitive deficits, memory loss, depression, and anxiety [4]. Although some of these symptoms may arise in response to living with a chronic disease, neuropsychiatric features can emerge prior to lupus diagnosis [5]. Due to its multifactorial nature, heterogeneous presentation, and unclear pathogenesis, diagnosing and managing NPSLE are two of the greatest challenges in providing care for lupus patients [4, 6].

Clinical studies have revealed clear evidence of neuroinflammation in NPSLE [7–9]. Lesions are frequently noted in white matter tracts and cortical atrophy is present [10, 11]. Increased intrathecal albumin concentrations and noninvasive imaging indicate that disruption of the blood–brain barrier and blood-cerebrospinal fluid (CSF) barrier occurs in NPSLE patients [7, 10, 12–14]. Autoantibodies, through effects such as opsonization and immune-complex formation, directly induce systemic features of lupus [15]. Brain-reactive autoantibodies, including those targeting the glutamatergic NMDA receptor of neurons, are significantly associated with NPSLE manifestations [4, 16, 17], and they appear potentially pathogenic in pre-clinical models [18, 19]. Furthermore, patient CSF contains elevated levels of these autoantibodies [20], as well as cytokines and other mediators of inflammation, including nitric oxide [21]. In the context of inflammatory mechanisms, however, interleukin-6 (IL-6) emerges as one of the most frequently elevated and promising markers of neuropsychiatric lupus.

While many SLE and NPSLE patients exhibit serum elevations in IL-6 [22, 23], higher levels can distinguish NPSLE patients [24]. Increased CSF concentrations of IL-6 show an even greater association with neuropsychiatric involvement [25–29]. Intrathecal IL-6 levels further correspond to markers of neuronal pathology, including demyelinating brainstem lesions [30] and CSF neurofilament levels [31]. Beyond impacting brain tissue directly, IL-6 upregulates brain barrier permeability, potentially promoting CNS entry of additional systemic neuroinflammatory molecules [32].

While homeostatic at low levels in the CNS, excessive IL-6 signaling can activate apoptotic pathways in neurons [33] and stimulate glial cell reactivity [34–38]. Microglia, the resident innate immune cells of the CNS, and astrocytes, key regulators of neuronal metabolism and CNS inflammation [39], are specifically implicated in NPSLE patients and animal models. Using diffusion imaging of key metabolites, damage to neurons and their axons was found to correlate with activation of glial cells [40].

High rates of microglial phagocytosis of synapses, the fundamental signaling connection between neurons, were observed in lupus mice [41]. Additionally, a perturbed genetic signature favoring neurodegeneration was found in lupus microglia [42]. Elevated levels of glial fibrillary acidic protein (GFAP), a marker of astrocyte activation and proliferation, have been found in the CSF of NPSLE patients [31]. In vitro, lupus mouse CSF was found to reduce the viability of neurons which were co-cultured with astrocytes, though it is unclear if the astrocytes directly mediated those neurotoxic effects [43]. Still, IL-6 activates and promotes the proliferation of astrocytes, a process called astrocytosis [38]. Through cumulative disruption of neuron health and function, glial reactivity, potentially under IL-6’s regulation, could be disrupting neurologic function in key brain regions to promote the neuropsychiatric disease associated with lupus.

Progress in understanding putative NPSLE etiologies has been notably slow due to the dearth of human tissue available for study. Therefore, and despite their limitations, mouse models have special importance in the investigation of this specific lupus manifestation, and they are commonly used for this purpose [44–53]. While modelling a complex and heterogeneous pathology like NPSLE in mice must be interpreted carefully, a large body of research demonstrates the immunologic, genetic, proteomic, and cellular overlap between the models and patient disease.

Pikman et al. previously highlighted some of these shared mechanisms, including B-cell activation, autoantibody production, complement-mediated endothelial damage, and cytokine upregulation. Lupus mice exhibit upregulated expression of both inflammatory (i.e., IL-17, IL-6) and anti-inflammatory (i.e., IL-10) cytokines in brain tissue [54], a finding readily seen in the periphery of lupus patients [55]. Nevertheless, little is known regarding the CSF composition of NPSLE mouse models. Establishing the composition of intrathecal fluid in an NPSLE model would enable the immediate subsequent assessment of the mechanistic impact of potential molecular mediators, using genetically manipulated mouse strains.

Perhaps the most widely used spontaneous lupus animal model, MRL/lpr mice replicate many of the serologic and organ manifestations of SLE, including elevated anti-dsDNA antibodies and lymphocyte-driven pathology in skin, kidneys, and brains [56]. Notably, the disease course is progressive in the MRL/lpr model without acute relapses or interval remissions. While thus limiting the ability of the MRL/lpr strain to model flares seen in human disease, it is still widely regarded as an excellent model of chronic disease processes leading to organ dysfunction [57]. Beyond systemic disease, MRL/lpr mice recreate many (albeit not all) neurologic manifestations of NPSLE [14, 51, 58–62], including serum and intrathecal elevations of brain-reactive autoantibodies (i.e., anti-NMDAR), inflammatory neuroendocrine interactions, hippocampal atrophy, breakdown in the brain’s barrier systems, and learning, memory, and affective deficits.

IL-6 levels increase early, typically by 5 weeks of age, in MRL/lpr mice, and an acute loss of sucrose preference (consistent with anhedonia) could be recreated in the MRL/mpj control strain by upregulating systemic IL-6 levels for 5 days using intraperitoneally delivered IL-6 [46, 63]. No impact of this transient intervention was observed on object-based memory tasks [47]. NPSLE patients often experience chronic memory issues independent of acute flares in activity [6], so chronic IL-6 exposure might instead mediate the learning and memory deficits modeled by MRL/lpr mice. The effects of long-term, rather than acute, IL-6 exposure on the behavioral deficits in MRL/lpr mice have not previously been carefully examined. Recently, a study in NZB/W-F1 lupus mice with behavioral deficits revealed that microglial activation in the hippocampus, a key brain region involved in memory and emotion, is associated with locally increased IL-6 levels [64]. Taken together, these findings tend to support the pathogenic potential of IL-6 and glia-mediated neurologic dysfunction in NPSLE.

The present study aimed to further uncover the role of IL-6 in NPSLE-like disease by testing the requirement for chronic IL-6 exposure in the behavioral features of MRL/lpr mice. To expand upon the clinical fidelity of this model, we additionally performed a high-throughput proteomic screen of the CSF of a lupus mouse model, and we discovered several inflammatory mediators, including IL-6, found also in CSF from human NPSLE patients. To establish the direct connection between chronic IL-6 exposure and the pathogenesis of NPSLE, we compared MRL/lpr mice with a constitutive knockout of the IL-6 gene to MRL/lpr mice with intact IL-6 expression. We assessed measures of systemic disease, the performance on neurobehavioral testing, and quantitative measures of glial pathology to test the hypothesis that constitutive IL-6 knockout MRL/lpr mice would exhibit improved behavioral features of NPSLE.

## Materials and methods

### Animals

**Fig. 1:**
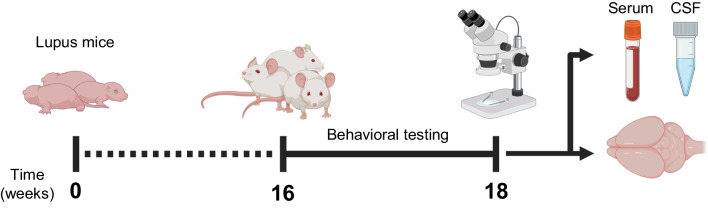
Experimental design schematic. Sixteen-week-old lupus or control mice underwent a validated behavioral testing battery over the course of two weeks. After testing, mice were sacrificed for collection of samples, including cerebrospinal fluid (CSF) from the cisterna magna, serum, and brain tissue

The experimental groups of interest included MRL/lpr lupus mice (Jackson Laboratory, #000485) and age-and-sex matched congenic control MRL/mpj mice (Jackson Laboratory, #000486). Additionally, we studied *il6*^−/−^ homozygous knockout MRL/lpr (IL-6 KO) mice and *il6*^+*/*+^ homozygous MRL/lpr control littermates (IL-6 WT). The former strain was originally generated by our collaborators at the University of Mainz [65]. Breeding pairs were transferred to the animal facility at the Albert Einstein College of Medicine (AECOM) where a multigeneration colony was successfully established. The zygosity of *il6* was determined in each weaned mouse by PCR. Once again, two separate cohorts (Additional file 1: Table S1; total n = 15 per genotype) were assessed using the same experimental timeline (Fig. 1).

For each genotype, we studied adult female mice of 14–18 (average sixteen) weeks of age. Female MRL/lpr mice exhibit accelerated and more prominent signs of lupus-like disease than do males [66] and display well established features of NPSLE by twelve weeks of age [61]. For these reasons, our group (and many other investigators) generally use female MRL/lpr mice [66, 67]. All animal husbandry and handling protocols were approved by the Institutional Animal Care and Use Committee at AECOM. Mice were locally housed in a specific pathogen free environment and were given ad libitum access to food and water.

### Behavioral testing

Standardized protocols were used to perform each of our behavioral tests, as described in full elsewhere [61, 68–70] (Additional file 1: Table S2). A two-week timeline for testing (Fig. 1) maximized experimental expediency considering the rapidly progressive lupus-like disease in MRL/lpr mice while respecting animal welfare concerns. All tests were performed and recorded using Viewer behavioral software (Biobserve). Automated quantification of time spent in spatially-gated zones was performed in each task; however, hand-timing is more accurate on behaviorally-nuanced tasks like object placement, object recognition, Porsolt swim, and social preference [68, 69, 71].

#### Behavioral spectrometry

To account for confounding variations in locomotor capability or activity level, mice were placed in an enclosed chamber (40 cm^2^) and their behavior was monitored using kinetic sensors in the floor, a digital camera, and automated animal behavior software (Viewer). Over a nine-minute trial, the software characterized movement track-length, time spent active, and frequency of behaviors like rearing, grooming, and alerting. Nine minutes provided ample time to detect abnormal behaviors, as previously published [71]. The presence of abnormal behavior, defined as absence of rearing or grooming, or insufficient activity, defined as low track length relative to the cohort, would have led to mice being excluded from further testing. These exclusion criteria were applied to minimize potential confounding effects of physical or behavioral hindrances on subsequent tasks. However, all mice in this study met the pre-specified inclusion criteria.

#### Open field task

In mice, time spent exploring the central portion of an open field is inversely correlated with anxiety-like behavior. During the behavioral spectrometry trial, an open field task simultaneously operated using Viewer software. The percentage of total trial time spent by the mouse within an 18 cm^2^ central area of the chamber’s floor was digitally quantified, with less center time reflecting anxiety-like behavior.

#### Object placement (OP) and object recognition (OR) tests

Mice have an innate preference to investigate new objects. This novelty preference was assessed using cognitive tasks dependent on spatial (OP) or recognition (OR) learning and memory. The same objects were used among all mice on each task. Objects were of equal visual saliency, of equivalent dimension, and sanitized to reduce inherent object preferences. These objects and protocols were rigorously validated by the Animal Behavior Core at AECOM and previously published [68, 69, 71, 72].

In both tasks, an initial training period involved placing mice in a field with two identical objects. Before the testing period, an intervening retention interval of 90 min (OP) or 120 min (OR) passed. For the testing trial, one object had been moved (OP) or replaced with a visually distinct object (OR). During testing, the ratio of time spent investigating the new object to the total time investigating both objects was recorded. The result was expressed as either a percentage or a group-wide failure rate. In those mice spending less than 55% of the time investigating the new object, respective learning and memory functions were defined as deficient [68, 69, 71].

#### Porsolt swim task

A validated measure of depression-like behavior, the Porsolt swim task involved placing mice into a transparent cylindrical tank filled to seventy-five percent capacity with 27 °C tap water. During a ten-minute observation period, the first minute was not scored to allow acclamation of the mice to the water. Three subsequent three-minute bins were scored. Increased amount of time spent immobile, expressed as a percentage of total time, reflected behavioral despair, a murine correlate of depression [61].

#### Social preference

Like the OP and OR tasks, mice were placed in a field containing two stimuli: another mouse behind a mesh barrier or an inanimate object. Normally behaving mice spend more time investigating the other mouse (socializing). During a single five-minute trial, the percent social preference was calculated. Again, a pass/fail threshold of 55% was used. In those mice which failed, a lack of social engagement, or social withdrawal, was attributed to affective features.

#### Elevated plus maze

Similar in principle to the open field task, mice with anxious behavior do not explore open areas. Each trial was ten minutes long, and mice were placed in a four-armed field. Two arms were enclosed, and two arms were open. The total amount of time in the open arm was recorded. Less time in the open arm corresponded to anxiety-like behavior.

### High-throughput proteomics

#### Microarrays

As previously described [73], serum and CSF samples were collected following behavioral testing from MRL/lpr and MRL/mpj mice at 18–19 weeks of age and interrogated for a total of 1308 protein antigens using the RayBio^®^ L-Series Mouse Antibody Array (RayBiotech, Catalog# AAM-BLG-1308–4).

#### Serum proteomics

Serum was collected at sacrifice and flash-frozen in liquid nitrogen. Individual 100 µL serum samples from eight MRL/lpr and eight MRL/mpj mice were processed on microarrays. The proteomic results of serum analysis have previously been published and reproduced with permission [73]. Significant markers were defined as those meeting multiple-test-corrected p-values < 0.05 and fold changes >|1.1|. The present study newly analyzed the relationship between the respective levels of array-measured serum IL-6 in the eight MRL/lpr mice and their scores on behavioral testing using Spearman rank correlations.

#### CSF proteomics

CSF was collected via cisternal puncture following intracardiac perfusion according to a previously described protocol [60]. Due to the minimal volume retrieved per mouse (10 µL), high-throughput analysis of individual mouse CSF samples was not feasible. To overcome this challenge, we pooled the CSF of ten mice of the same genotype to produce a single 100 µL sample. Each pooled, 100 µL sample was further diluted fourfold to provide sufficient volume for serial incubation on each of the three slides comprising the 1308-plex protein microarray, as recommended by the array manufacturer. Three pooled samples were collected for MRL/lpr mice and three for MRL/mpj mice. These six samples were run on two 4-sample array kits, and the results were normalized to internal standards to enable cross-experiment combining of data. A modified significance threshold of p < 0.1 was chosen because of our  pre-existing expectation of detecting proteins seen in human NPSLE, and due to the resulting limited sample size in the CSF microarray analyses. This accommodation was warranted as these exploratory analyses sought to compare the MRL/lpr CSF composition to human findings.

#### ELISA validation of serum IL-6

We measured serum concentrations of IL-6 in MRL/lpr and MRL/mpj mice from the first and second cohorts described above (which were not part of the serum array studies). Quantification was performed using the mouse IL-6 Quantikine ELISA kit (R&D Systems, Minneapolis, MN; Cat# M6000B) as per manufacturer instructions.

### Systemic disease assessment in IL-6 KO and IL-6 WT mice

IL-6 KO mice were compared to IL-6 WT mice in the extent of systemic lupus-like disease. Previously, the development of lymphoproliferative and renal disease has been shown to be delayed in IL-6 KO MRL/lpr mice [65]. We performed repeat assessments of these features both to validate the phenotype of these mice in our local facility and to determine if behavioral changes correspond with indicators of systemic disease.

#### Lymphoproliferative disease

Lymphoproliferation, which reflects the expansion of pathogenic T- and B-cells in lupus, manifests as splenomegaly and lymph node enlargement [15]. Lymphadenopathy was scored by palpation of cervical, axillary, and inguinal lymph nodes bilaterally (score of 1: a single node could be felt, 2: multiple nodes on one side, 3: moderate multiple bilateral nodes, 4: significant multiple bilateral nodes). Splenomegaly was measured by weighing the spleen of each mouse following sacrifice.

#### Renal disease

Using ELISA [74], serum levels of blood urea nitrogen (BUN) were measured to assess renal function. Higher serum BUN indicates worse renal function [75, 76].

#### Humoral disease

Anti-double stranded DNA (anti-dsDNA) antibodies were measured in the serum of the IL-6 KO and IL-6 WT mice by ELISA, as described [71, 77].

### Bulk RNA expression

From each mouse, we collected one whole hemisphere of cortex from its complete rostral to caudal extent and unilateral hippocampal tissue. We then performed real-time quantitative polymerase chain reaction (qPCR) analysis following RNA isolation, cDNA synthesis, and qPCR protocols previously published [68]. Inclusion of sample data in the experiment was determined prior to expression analysis based on standard measures of optimal reaction quality (exclusion criteria: multiple peaks, reaction failure, between replicate variance). No post-hoc criteria were used to select samples included in the qPCR analysis. The delta-delta CT method was used to quantify gene expression (housekeeping: *ywhaz*; reference tissue: MRL/mpj cortex). Genes assessed included *aif1* (Iba-1, microglia), *gfap* (astrocytes), and *nos2* (nitric oxide synthase)*.* Elevated *nos2* expression is a marker of inflammatory response by microglia [78].

### Histologic quantification of glial cells

#### Immunofluorescent imaging

Brains were collected, paraffin-embedded, stained, and imaged as previously described [68]. Brains were sectioned at two rostral-caudal levels (third-ventricle and hippocampus: bregma − 2.2 mm; rostral mesencephalon: bregma—4 mm). Separate slides were used to stain microglia and astrocytes. Nuclei were stained using DAPI. Microglial labeling used a rabbit-anti-mouse Iba-1 primary antibody (1:100; Fujifilm, Richmond, VA; Catalog# 19–19,741). Astrocytes were labelled using a rabbit-anti-mouse GFAP primary antibody (1:100; Invitrogen, Waltham, MA; Catalog #13–0300). Corresponding Alexa-Fluor-488 tagged secondary antibodies were used (1:200). Cellular apoptosis was assessed in brain sections using the ApopTag TUNEL-detection kit (Millipore, Catalog #S7110).

Iba-1 and GFAP stained slides were separately imaged and analyzed. Slides were de-identified so that researchers were blind to the genotype of the mouse being imaged and analyzed. An EVOS Fl auto 2 automated fluorescent microscope was used to image cortical and subcortical brain regions which were functionally relevant to learning, memory, or navigation.

#### Regions of interest

Via automated cell-counting with standardized cell-body detection thresholds, ImageJ software [79] was used to quantify the density per square millimeter of Iba-1 positive or GFAP positive cells in each region of interest. Regarding the subcortical structures investigated, the hippocampus is fundamental to learning and memory. The dentate gyrus was prioritized as it is a key mediator of these functions, the primary input for cortical information, and exhibits microglia-related pathology in MRL/lpr mice [41, 80]. The amygdala also plays a central role in motivated behavior and conditioning-based memory [80].

The somatosensory cortex was studied for its role in sensory processing and spatial navigation in object based behavioral tasks [81]. We quantified glial density in the retrosplenial cortex because this region has roles in spatial memory and can influence performance on our animal-cognition tasks [82, 83]. Similarly, the entorhinal cortex is a pivotal component of cortical and hippocampal interactions which influences novelty-based performance [83]. As a result, the entorhinal cortex can modulate the functionality of this network which is vital for learning and memory. In the mouse, the entorhinal region is in the caudal and inferior pole of the cortex.

Mouse brain atlases were used to identify brain regions based on relative location to prominent landmarks [84]. Four brain sections were labeled and imaged for each mouse, two Iba1 (rostral + caudal sections) and two GFAP (rostral + caudal sections). Single field images (1 mm^2^) were collected from each labeled rostral brain section for the retrosplenial cortex, somatosensory cortex, amygdala, and hippocampal dentate gyrus. Separately, two caudal brain sections were imaged to capture the more distal entorhinal cortex, again one for each glial marker. The rhinal fissure was used as the superolateral boundary of the entorhinal cortex, external capsule as the medial boundary, and the capsule was followed to the inferomedial pole of the cortex. Two to five images were collected and stitched together of each entorhinal cortex to capture this area.

### Statistical analyses

A two-sided Chi-square analysis was performed for all pass/fail tests. The 1.5*IQR technique was used to identify outlier values. The Jarque–Bera method was then used to assess normality in all datasets, and appropriate two-tail parametric (Students T-test with Welch’s correction) or non-parametric (Mann Whitney U test) means-based comparisons were used accordingly (p < 0.05 is significant). Ordinal data was assessed with Mann Whitney U test as well. Of note, our analysis of histologic data was performed with one-tail T-tests, because we hypothesized that cortical glial cells would be decreased in the IL-6 KO, given the findings in the gene expression analysis. This evidence-based assumption of directionality validates the use of one-tail statistical testing. GraphPad Prism 9 and Microsoft Excel were used to perform all statistical analyses and produce all graphs.

## Results

### Proteomic analyses of MRL/lpr serum and CSF implicate IL-6

**Fig. 2:**
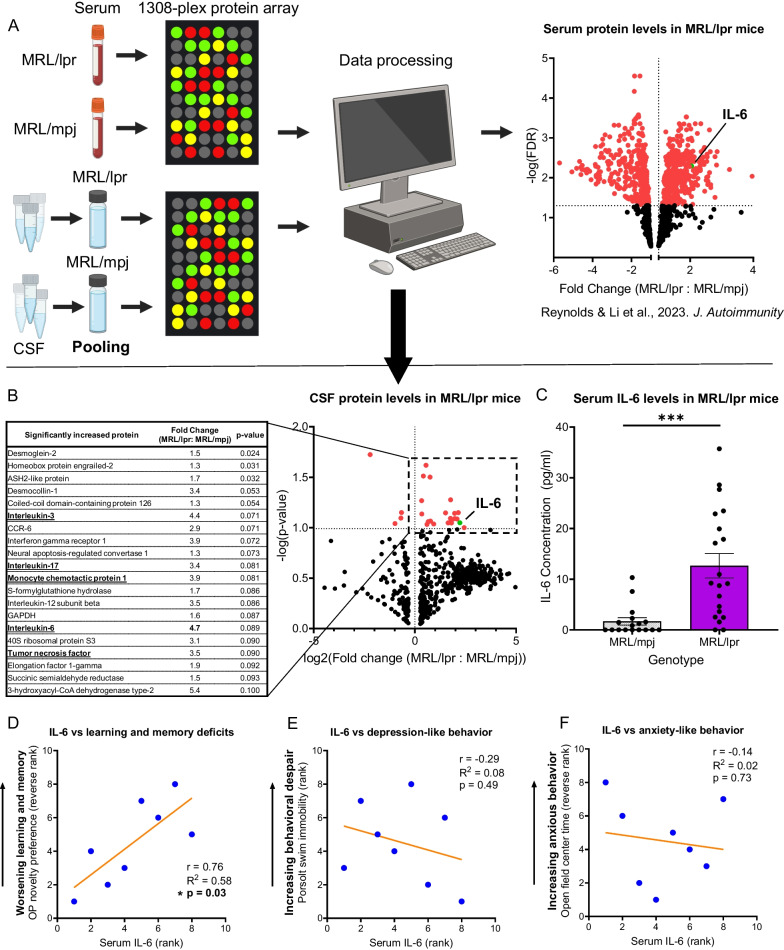
Elevated IL-6 in MRL/lpr mice correlates with learning and memory dysfunction. **A** To identify potentially pathogenic molecules in NPSLE, high-throughput proteomic screening of MRL/lpr serum and CSF relative to MRL/mpj samples was performed using RayBiotech 1308-plex mouse protein microarrays. The serum findings (previously published and reproduced here with the permission of the *Journal of Autoimmunity*) indicated that interleukin-6 (IL-6) is significantly increased in the serum of MRL/lpr mice. **B** Ten to fifteen CSF samples from either mouse strain were pooled to yield a single 100 µl analyte. This process was repeated twice more, so that protein content of six separate pools (three MRL/lpr and three MRL/mpj) was analyzed using the arrays. A volcano plot is shown, depicting the CSF proteomic array results. Significantly different proteins, including IL-6, are highlighted in red and summarized in the table (left). Of note, neural apoptosis-regulated convertase, GAPDH, and succinic semialdehyde reductase have each been linked to neurotoxicity. Inflammatory cytokines with known roles in human lupus, including IL-3, IL-6, IL-17, MCP-1, and TNF-α, were increased as well (bold, underlined). **C** Increases in IL-6 previously found on the protein array were validated by measuring IL-6 in the serum of independent cohorts of MRL/mpj vs MRL/lpr mice using ELISA. **D**–**F** Using Spearman rank correlations, array-measured serum IL-6 levels from individual MRL/lpr mice were assessed for relationship with performance on object placement (OP, **D** learning and memory deficits), Porsolt swim (**E** behavioral despair), and open field (**F** anxious behavior) tasks. On OP and open field tasks, lower scores were associated with more severe disease. To better represent the correlation between increasing serum IL-6 and worsening disease, the score-based ranks for these tests were plotted in reverse order, with higher ranks representing mice with lower novelty preference/center time (worse performance). *p < 0.05; ***p < 0.0005

Following a standard experimental timeline (Fig. 1), we performed behavioral testing and collected samples from a large cohort of MRL/lpr and control MRL/mpj mice (n = 40 per genotype). Having confirmed the presence of the expected behavioral features (Additional file 1: Data S1), we performed a 1308-plex proteomic screening of serum from eight MRL/lpr and eight MRL/mpj mice (Fig. 2A, top). The serum proteome profile and the relationship to systemic, rather than neuropsychiatric, disease have been previously published [73]. However, the presence of IL-6 and it’s correlation with behavior had not been previously analyzed. Separately, we conducted screening of pooled CSF samples from MRL/lpr or MRL/mpj mice (n = 3 pools per genotype; Fig. 2A, bottom). In the CSF of MRL/lpr mice, we found twenty proteins increased above levels in MRL/mpj mice (Fig. 2B). Among these molecules, which included IL-3, IL-17, MCP-1, and TNF-α, IL-6 had one of the highest fold increases (FC = 4.7).

Previously, we reported that IL-6 is elevated in MRL/lpr serum by protein array [73]. We now assessed the relationship of IL-6 with performance on specific behavioral tasks (Fig. 2D–F). Decreasing novelty preference on behavioral testing reflects increasing learning and memory deficits in mice. Worsening learning and memory on the OP task was found to strongly correlate with serum IL-6 level (r = 0.76, R^2^ = 0.58, n = 8, p = 0.03). Neither Porsolt swim nor open field performance correlated, however, with serum IL-6 levels. Additionally, no correlation was found between performance on any behavioral test and serum levels of total IgG or anti-dsDNA autoantibody levels in this cohort of mice.

To confirm an increase in serum IL-6 and assess the change more quantitatively, we studied serum IL-6 levels in independent cohorts of MRL/lpr and control mice by ELISA (Fig. 2C). IL-6 was indeed significantly increased in lupus mouse serum (MRL/lpr: 12.6 ± 2.4 pg/mL (n = 20) vs MRL/mpj: 1.7 ± 0.7 pg/mL (n = 17); ***p = 0.0003).

### IL-6 KO MRL/lpr mice display improved learning and memory

**Fig. 3:**
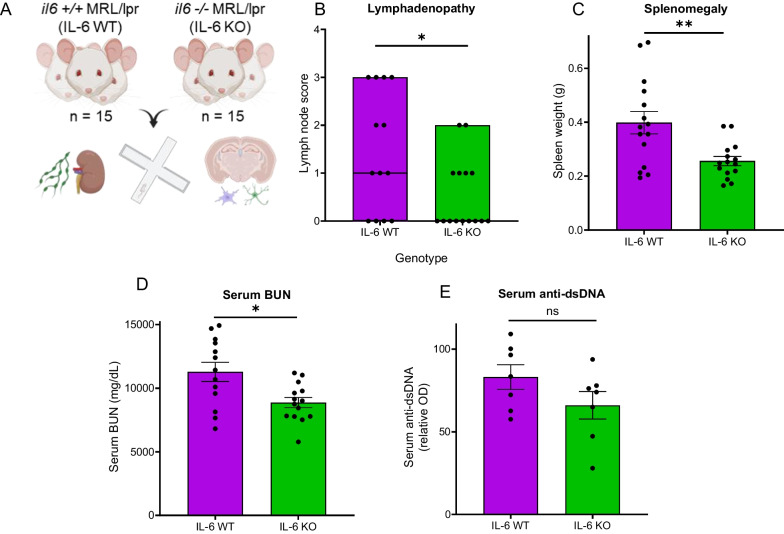
IL-6 KO MRL/lpr mice show reduced lymphoproliferation and renal disease. **A** MRL/lpr mice with intact (*il6*^+/+^ ; IL-6 WT) IL-6 expression were compared to MRL/lpr mice with a genetic deletion of IL-6 (*il6*^−/−^; IL-6 KO) for signs of systemic lupus activity. Two separate cohorts (cohort A: 7 mice per genotype; cohort B: 8 mice per genotype) were used to confirm reproducibility. **B** Lymph node scores were determined by palpability of cervical, axillary, and inguinal lymph nodes at sacrifice. Higher scores equate to larger nodes in multiple locations. Horizontal line on bars reflects the median (IL-6 KO median is 0 and is superimposed on x-axis). **C** Enlargement of the spleen, or splenomegaly, is another feature of lymphoproliferation in the MRL/lpr strain which was quantitated by weighing the spleen following sacrifice. **D** Renal disease, a key feature of systemic lupus, was assessed by measuring serum blood-urea nitrogen (BUN). Increased levels of BUN reflect poorer renal function. **E** Serum titers of antibodies targeting double-stranded DNA (anti-dsDNA), a key marker of lupus serological disease activity, were measured using ELISA. *p < 0.05; **p < 0.005

By comparing MRL/lpr mice devoid of IL-6 expression (*il6*^−/−^; IL-6 KO) to those with intact IL-6 (*il6*^+/+^ ; IL-6 WT), we were able to assess changes in systemic and neuropsychiatric lupus-like disease regulated by IL-6 (Fig. 3A). IL-6 KO mice displayed reduced lymphoproliferative (Fig. 3B: *p = 0.049; 3C: **p = 0.005) and renal (Fig. 3D: *p = 0.01) disease compared to age-matched IL-6 WT, as reported previously [65]. Serum anti-dsDNA level did not differ between genotypes (Fig. 3E).

**Fig. 4:**
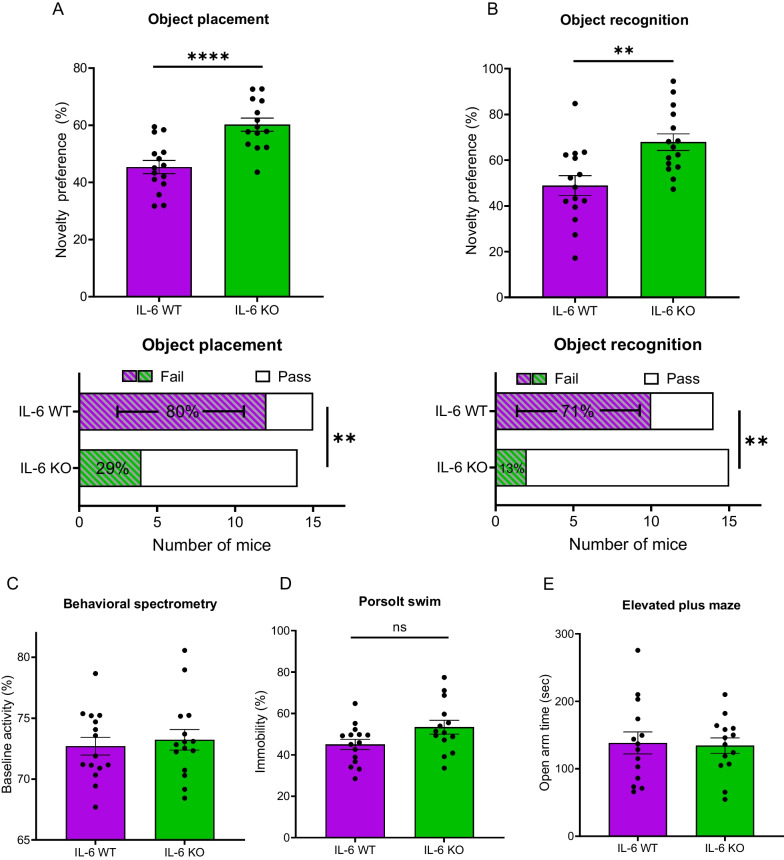
Position and identification memory scores were higher in IL-6 KO MRL/lpr mice. Neuropsychiatric disease was assessed in IL-6 WT and IL-6 KO mice to determine the contribution of IL-6 to the behavioral features of the disease. **A** Performance on the object placement task is reported as a comparison of percent novelty preferences (top) or failure rates (bottom) in both genotypes. **B** Similarly, results of IL-6 WT vs IL-6 KO comparison on the object recognition task are presented in both continuous (top) and failure rate (bottom) forms. **C** Baseline activity on behavioral spectrometry was compared between genotypes to assess for the presence of any fundamental differences in locomotion or other confounding features. IL-6 WT comparisons to IL-6 KO also included testing for affective features using the following tasks: Porsolt swim (**D**; immobility to reflect behavioral despair), elevated plus maze (E; exploration of open area to determine anxiety-like behavior), and social preference task (data not shown). **p < 0.005; ****p < 0.0001

On behavioral testing, IL-6 KO mice displayed higher novelty preference scores than WT mice on OP (Fig. 4A, top; IL-6 KO: 60.2 ± 2.3% (n = 14) vs IL-6 WT: 45.4 ± 2.3% (n = 15); ****p < 0.0001). Additionally, the failure rate on OP was significantly reduced in IL-6 KO mice (Fig. 4A, bottom; IL-6 KO: 29% (n = 14) vs IL-6 WT: 80% (n = 15); **p = 0.005). Similarly, IL-6 deficient mice displayed higher novelty preference on the OR task than those MRL/lpr mice with intact IL-6 expression (Fig. 4B, top; IL-6 KO: 67.9 ± 3.6% (n = 15) vs IL-6 WT: 48.9 ± 4.3% (n = 15); **p = 0.002). Concordantly, fewer IL-6 KO failed OR compared to wildtype mice (Fig. 4B, bottom; IL-6 KO: 13% (n = 15) vs IL-6 WT: 71% (n = 14); **p = 0.002).

Using behavioral spectrometry, no differences were found in baseline activity or locomotion between IL-6 KO and IL-6 WT mice (Fig. 4C). Regarding assessment of behavioral despair, immobility rates on the Porsolt swim test did not differ by genotype (Fig. 4D; IL-6 KO: 53.4 ± 3.3% (n = 14) vs IL-6 WT: 45.0 ± 2.5% (n = 15); p = 0.054). IL-6 KO mice also spent comparable amounts of time exploring the open arms on elevated plus maze (Fig. 4E) and did not exhibit altered social withdrawal (data not shown). Behavioral testing results are summarized in Table 1.

**Table 1:** Behavioral testing results

Condition	Genotype	n	Cohort	OP novelty preference (%)	OR novelty preference (%)
IL-6 WT	IL-6 + / + MRL/lpr	14–15	A + B	45.4 ± 2.3	48.9 ± 4.3
IL-6 KO	IL-6 -/- MRL/lpr	14–15	A + B	60.2 ± 2.3	67.9 ± 3.6
p-value	–	–		**9.7 × 10**^–5^****	0.002**

Condition	Behavioral spectrometer (% activity)	PS immobility (%)	Open field time in center (% of total)	EPM open arm (seconds)	SP (%)
IL-6 WT	72.7 ± 0.7	45.0 ± 2.5	15.5 ± 1.7	138.3 ± 16.3	53.1 ± 2.3
IL-6 KO	73.2 ± 0.8	53.4 ± 3.3	18 ± 2.3	134.4 ± 11.4	54 ± 2.1
p-value	0.647	0.054	0.380	0.436	0.783

IL-6 KO vs IL-6 WT scores in behavioral testing. Means ± standard errors are provided for each outcome measured. Within-genotype outcomes were statistically equivalent between cohort A and B

OP, object placement; OR, object recognition; PS, Porsolt swim; EPM, elevated plus maze; SP, social preference

Bold p-values are significant. **p < 0.01, ****p < 0.0001

### Gliosis is reduced in the IL-6 KO cortex

Having detected IL-6 dependent differences in learning and memory, we aimed to uncover the potential mechanism driving this effect in lupus mice. Each of the IL-6 KO/WT cohorts was allocated separately to qPCR (cohort A) or histologic (cohort B) analysis of the brain. Given the significant inflammatory CSF profile in lupus patients which we now had confirmed in the MRL/lpr strain, we assessed gliosis in key brain regions as a measure of this neuroinflammatory signature.

**Fig. 5:**
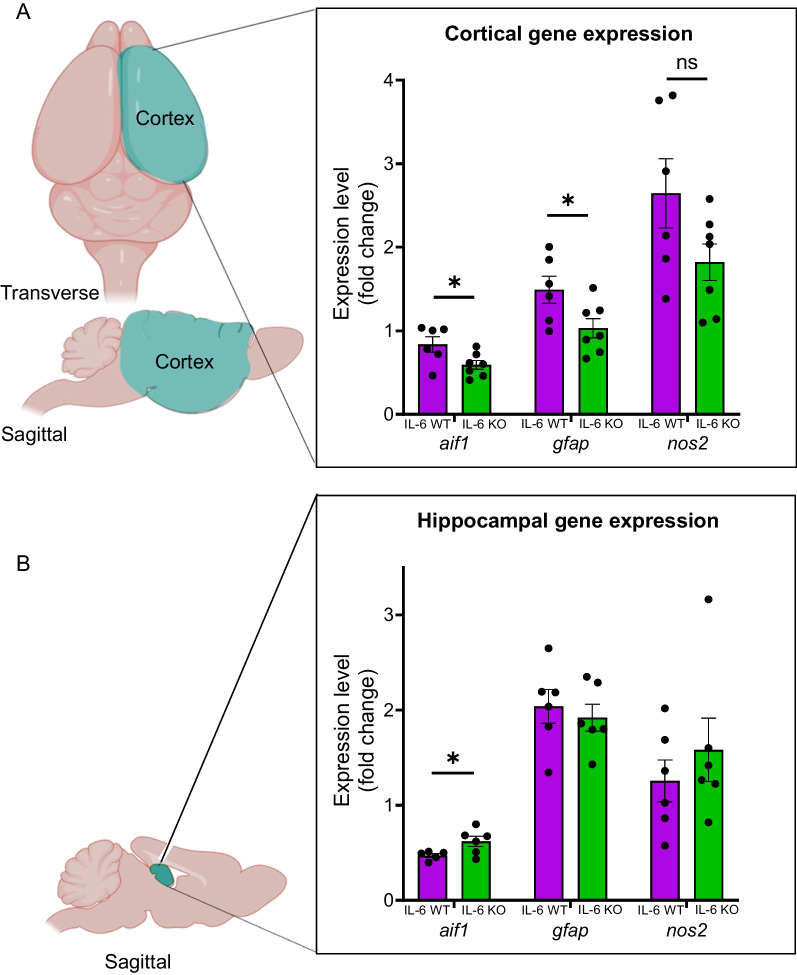
Cortical expression of glial genes is reduced in IL-6 KO MRL/lpr mice. Bulk gene expression was quantified in a whole-hemisphere of cortex (**A**; blue region on diagram) and the unilateral hippocampus (**B**; blue region on diagram) from IL-6 KO mice and compared to that of IL-6 WT mice. The reference sample was cortex, collected from a single sex and age-matched MRL/mpj mouse. One of the two separate cohorts (Cohort A) was allocated to quantification of brain gene expression using real time quantitative PCR, while the other (Cohort B) was reserved for subsequent histologic analysis. Measured genes included *aif1* (allograft inhibitor factor 1; Iba-1 protein) which reflects monocytic and microglial activation/proliferation, *gfap* (glial fibrillary acidic protein) which reflects astrocytic activation/proliferation, and *nos2* (nitric oxide synthase 2) which is a producer of inflammatory nitric oxide. *p < 0.05

Unilateral whole cortex and hippocampus samples were dissected from the brain of each mouse and evaluated for inflammatory gene expression using qPCR. Cortical expression, measured in fold change (FC), of the monocyte and microglia marker *aif1* (Iba-1) was reduced in IL-6 KO mice compared to IL-6 WT controls (Fig. 5A; IL-6 KO: 0.59 ± 0.05 FC (n = 7) vs IL-6 WT: 0.84 ± 0.09 (n = 6); *p = 0.0499). Similarly, cortical expression of the astrocyte marker *gfap* was reduced in IL-6 deficient mice (Fig. 5A; IL-6 KO: 1.03 ± 0.11 FC (n = 7) vs IL-6 WT: 1.49 ± 0.16 (n = 6); *p = 0.044). No difference was found in cortical expression of *nos2*, a marker of nitric oxide synthetic activity (Fig. 5A; IL-6 KO: 1.82 ± 0.22 FC (n = 7) vs IL-6 WT: 2.65 ± 0.41 (n = 6); p = 0.118). In the hippocampus, IL-6 KO mice displayed elevated *aif1* expression relative to wildtype mice (Fig. 5B; IL-6 KO: 0.62 ± 0.05 FC (n = 6) vs IL-6 WT: 0.47 ± 0.02 (n = 5); *p = 0.04). Neither *gfap* nor *nos2* expression in the hippocampus differed between genotypes (Fig. 5B). Additional file 1: Table S3 summarizes the cortical and hippocampal gene expression analysis.

**Fig. 6:**
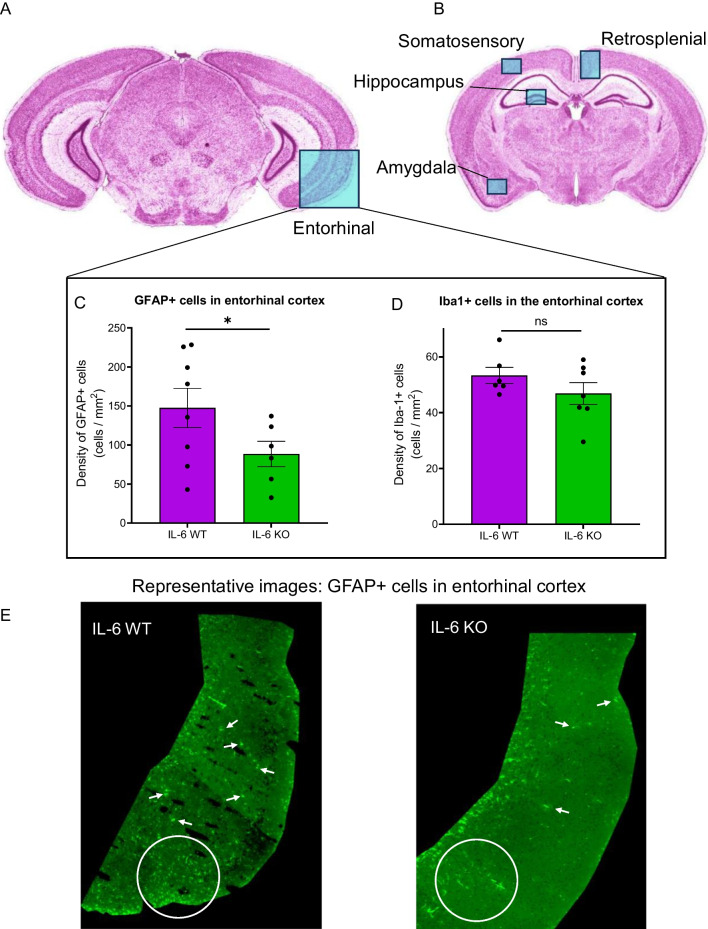
Density of astrocytes is reduced in the IL-6 KO MRL/lpr entorhinal cortex. Given potential cortical and hippocampal glial changes in the presence of IL-6, we performed immunofluorescent histologic analysis of astrocyte (GFAP + cells) and microglia (Iba + cells) numbers in the brains of IL6 KO relative to IL-6 WT lupus mice belonging to Cohort B. **A**, **B** The two-dimensional density of both cell types was assessed in cross-sections of the entorhinal cortex, somatosensory cortex, retrosplenial cortex, amygdala, and hippocampal dentate gyrus. **C**, **D** Comparisons of the density of GFAP + cells (astrocytes) and Iba1 + cells (microglia) between the entorhinal cortical regions of IL-6 WT and IL-6 KO mice. **E** Representative images of GFAP staining in the entorhinal cortices of IL-6 WT (left) and IL-6 KO (right) mice. Arrows point to a random sampling of stained cells. Coronal brain sections in panels **A** and **B** were obtained from the Harvard High Resolution Mouse Brain Atlas published by Sidman et al. [113]. *p < 0.05

Having detected cortical decreases in microglial and astrocytic gene expression, we used immunofluorescence to determine the specific region(s) responsible for those findings in IL-6 KO mice. Additionally, we sought to corroborate any change in microglia in the hippocampus. We quantified Iba1-positive (Iba1 + ; microglia) and GFAP-positive (GFAP + ; astrocytes) cells in the entorhinal cortex (Fig. 6A) and multiple other cortical and sub-cortical regions (Fig. 6B), including the somatosensory cortex, retrosplenial cortex, amygdala, and dentate gyrus of the hippocampus. Within the entorhinal cortex, we observed significantly reduced density of GFAP + cells in IL-6 KO mice relative to IL-6 WT mice (Fig. 6C; IL-6 KO: 89 ± 16 cells/mm^2^ (n = 6) vs IL-6 WT: 148 ± 25 cells/mm^2^ (n = 8); * p = 0.037). No significant difference was found in the density of Iba1 + cells (Fig. 6D; IL-6 KO: 47 ± 4 cells/mm^2^ (n = 7) vs IL-6 WT: 53 ± 3 cells/mm^2^ (n = 6); p = 0.11). Representative GFAP-stained images of the entorhinal cortex (Fig. 6E) from IL-6 WT (left) and IL-6 KO mice (right) demonstrate the quality of staining and distribution of cells.

The observed *aif1* expression increase in the IL-6 KO hippocampus did not correspond to altered density of Iba1 + cells in the hippocampal dentate gyrus (IL-6 KO: 12.0 ± 0.9 cells/mm^2^ (n = 8) vs IL-6 WT: 10.1 ± 0.8 cells/mm^2^ (n = 8); p = 0.07). Exploration of the CA1 region also did not reveal Iba1 + cell density changes, nor did it detect changes in CA1 thickness (Additional file 1: Data S2). No density differences in either glial cell subtype were found within the remaining cortical and sub-cortical regions. Similarly, the number of TUNEL-positive apoptotic cells was comparable in both the hippocampus and entorhinal cortex of IL-6 KO and IL-6 WT mice (2–5 cells per region)(data not shown). Table 2 provides glial density results.

**Table 2:** Histologic glial cell quantification in the brain

Condition	Genotype	GFAP + cells in s.sensory cx	GFAP + cells in amygdala	GFAP + cells in dentate gyrus	GFAP + cells in entorhinal cx
IL-6 WT	IL-6^+/+^ MRL/lpr	15 ± 7	57 ± 18	125 ± 25	148 ± 25
IL-6 KO	IL-6^−/−^ MRL/lpr	10 ± 2	37 ± 9	140 ± 45	89 ± 16
p-value	**–**	0.476	0.172	0.211	0.037*

Condition	Iba1 + cells in retrosplenial cx	Iba1 + cells in s.sensory cx	Iba1 + cells in amygdala	Iba1 + cells in dentate gyrus	Iba1 + cells in entorhinal cx
IL-6 WT	28 ± 2	21 ± 1	28 ± 2	10 ± 1	53 ± 3
IL-6 KO	37 ± 6	24 ± 1	28 ± 1	12 ± 1	47 ± 4
p-value	0.092	0.500	0.396	0.070	0.106

Quantification of the density (cells/mm^2^) of GFAP + and Iba1 + cells in key brain regions. Cohort A was used for gene expression analyses, Cohort B was used for histologic analysis. Mean density ± standard error for each outcome measured. Within-genotype outcomes were statistically equivalent

cx, cortex; s.sensory, somatosensory

Bold p-values are significant. *p < 0.05

## Discussion

Prior literature had indicated IL-6’s relationship to human NPSLE [4, 7, 22, 23, 31], but little was known regarding the pathogenic relevance of long-term IL-6 exposure in animal models of neuropsychiatric lupus. Moreover, existing knowledge was limited regarding the CSF proteome of the MRL/lpr mouse strain, which is the most widely studied murine model of NPSLE. In the present study, we found elevated IL-6 concentrations in the CSF of MRL/lpr mice and that serum IL-6 levels correlated with learning and memory abnormalities. Moreover, deficits in these particular neurologic functions were significantly improved in IL-6 KO MRL/lpr mice. Furthermore, astrocytosis was decreased in the IL-6 KO entorhinal cortex, a brain region vital to cognition, learning, and memory. Taken together, these findings demonstrate an instrumental role of IL-6 in the pathogenesis of NPSLE.

### Inflammatory CSF composition in MRL/lpr mice

NPSLE patients experience memory deficits, depression, and anxiety, and animal correlates of each feature have previously been described in MRL/lpr mice [59, 61]. Due to these attributes, the MRL/lpr mouse has long been used to model NPSLE. However, the degree to which the overall protein composition of murine CSF resembled that of human lupus was unknown. We took a meaningful step toward clarifying this ambiguity by using a multiplex protein array screening protocol to interrogate over 1300 proteins in the CSF of MRL/lpr mice with validated behavioral deficits.

This approach, however, was limited by the minimal volume of CSF available per mouse which required pooling of CSF from many mice. Additionally, we were limited to only three pooled analytes due to the resources required (i.e., forty mice per genotype to produce three pooled samples). We also expected, a priori, increased IL-6 concentrations in MRL/lpr CSF, given the many similarities between this model and human SLE. Therefore, we adopted a modified significance threshold of p < 0.1 that, while justified in this case, can be considered a limitation of our study. The primary consequences of these technical limitations are the less robust statistical significance of this particular experiment and the detection of only twenty increased proteins. Additionally, pooled techniques, while having been required for the high-throughput analyses, can potentially mask individual variations among mice, a possible constraint when studying a heterogeneous disease like SLE. Future studies could improve on this method by serially collecting larger volumes of CSF over time so that samples from individual mice may be analyzed. Development of such a method is currently in progress in the laboratory. Nonetheless, the pooled approach facilitated our primary goal which was to compare the CSF composition of the MRL/lpr model as a whole to existing clinical reports of the CSF composition in NPSLE patients.

Among the twenty proteins we found to be increased in MRL/lpr CSF, multiple inflammatory cytokines were present, including IL-3, IL-17, MCP-1, TNF-α, and IL-6. Previously, IL-3 serum levels in lupus patients were found to correlate with the interferon signature, a cytokine considered a primary driver of SLE [85]. However, IL-3 has not yet been identified in patient CSF. IL-17, a potent stimulator of autoimmunity, is increased in the CSF of NPSLE patients relative to non-lupus patients [86]. Similarly, TNF-α, a key systemic and neuroinflammatory mediator, is increased in human lupus CSF [9], as is MCP-1, which attracts monocytes and microglia to sites of inflammation [87]. The composition of the MRL/lpr CSF, therefore, contained several cytokines observed in NPSLE patients’ CSF, adding validity to the model and enhancing the translational relevance of our results.

Regional gray matter atrophy is observed in the brains of NPSLE patients and MRL/lpr mice [10, 11, 31, 88]. The CSF of MRL/lpr mice also contained proteins indicative of ongoing neuronal loss. Neural apoptosis-regulated convertase 1 is upregulated in neurons undergoing apoptosis, and increased levels have been shown within the brains of patients with neurodegenerative diseases [89]. Similarly, increased CSF content of glyceraldehyde-3-phosphate dehydrogenase is linked with the death of neurons in degenerative disease [89]. Furthermore, succinic semialdehyde reductase was shown to increase in the brain in response to oxidative stress, and increased glial expression has been associated with the loss of neurons [90]. Among all these findings, however, the elevation of IL-6 perhaps most faithfully replicates patient findings, and therefore it was the chosen focus of this particular study. IL-6 is often regarded to be the CSF cytokine most associated with NPSLE [4, 87], and its presence in MRL/lpr mice supported our subsequent investigations.

While not addressed by the present study, identifying the origin of intrathecal inflammatory markers, including IL-6, is an active focus of lupus research [91, 92]. IL-6 could gain entry to the CNS from the circulation by disruption of the brain’s barrier systems [62, 68, 93]. Alternatively, CNS resident glia and neurons could produce the IL-6 detected in the CSF [38, 94]. Prior work has indicated that NPSLE-like disease persists despite near-total suppression of systemic inflammatory activity [70]. A separate CNS pathology appears to develop, likely early in SLE progression [5, 64]. Conversely, short term augmentation of IL-6 levels systemically was sufficient to acutely induce anhedonia in non-lupus mice [46, 47]. Follow up studies aimed at determining whether IL-6 enters the brain from the periphery or is secreted by reactive resident brain cells (or both) would likely lend valuable mechanistic insight to our findings. In any case, the focus of our study was to determine if global IL-6 production mediates the features of NPSLE.

### IL-6 correlates with and is necessary for learning and memory deficits in MRL/lpr mice

Prior to this study, it was not known if murine NPSLE would develop in the constitutive absence of IL-6. Through proteomic analyses, we detected serum and CSF elevations of IL-6 in MRL/lpr mice. We further found that serum IL-6 levels correlated with worse learning and memory function without influencing affective features. Beyond IL-6, autoantibodies likely contribute to the pathogenesis of NPSLE-like disease in MRL/lpr mice [17, 19, 91]. While we did not find a correlation between serum antibody titers and behavioral features, future studies are needed to determine the interplay between IL-6 and brain-reactive autoantibodies in NPSLE. For example, the deposition of immunoglobulins in brain tissue could be assessed for its potential inflammatory capacity and contribution to behavioral deficits in MRL/lpr mice. Nonetheless, our initial CSF findings and serum correlations emphasized the importance of focusing on IL-6 in this study.

Our study replicated the decreases in lymphoproliferation and renal function seen in the original IL-6 KO MRL/lpr study [65]. IL-6 potently stimulates T- and B-cell proliferation [95], likely explaining depressed lymphoproliferation in IL-6 KO mice. Reduction in the number of lymphocytes, a cell type which drives the pathogenesis of lupus nephritis [15], could then explain the concurrent improvement in renal disease. However, as IL-6 stimulates antibody production by B-cells [96], serum anti-dsDNA titers were surprisingly unchanged. Compensatory signals, such as IL-4 or IFN-γ, could possibly continue to stimulate humoral immunity in the absence of IL-6 [96].

IL-6 KO mice showed improvement in learning and memory with unaltered murine correlates of depression or anxiety. Behavioral results between our two IL-6 KO/WT cohorts were highly comparable within each genotype, strengthening the reproducible nature of our findings. Nonetheless, several additional limitations of our experimental design should be mentioned. MRL/lpr mice replicate the hyposmia seen in some lupus patients [48, 97]. While it is possible that this reduced olfactory perception can influence performance on behavioral tasks, hyposmia in lupus patients is significantly associated with male sex [98]. Furthermore, the MRL/lpr studies by Kapadia et al. [48, 97] which validated this olfactory deficit used only male mice. We contend that our behavioral experiments, which used exclusively female mice because of their earlier modelling of severe disease [66], were much less likely to be confounded by hyposmia. Nonetheless, a formal evaluation of an olfactory phenotype in the IL-6 knockout strain might be of interest. While MRL/lpr mice model chronic features of lupus with high fidelity, this strain (and other lupus animal models as well) does not exhibit the acute disease exacerbations (flares) that can be seen in NPSLE patients. Our findings, then, better reflect the contribution of IL-6 to the lifelong deficits in memory performance described by many patients [1].

An MRL/mpj control group was not included in the IL-6 KO/WT cohorts for behavioral testing due to the comprehensive existing characterizations of this strain relative to IL-6 competent MLR/lpr mice. While inclusion of this group might have been ideal, we did find that knocking out IL-6 was associated with a higher novelty preference (+ 25% preference) relative to MRL/lpr mice that was comparable to historical MRL/mpj controls (+ 10–30% preference) [99, 100]. Therefore, abrogation of IL-6 signaling appears to return the memory and learning functions of MRL/lpr mice to those of non-lupus controls, at least in this indirect comparison. Inclusion of IL-6 KO MRL/mpj control mice, while not currently available, could help validate the specific effect of IL-6 on lupus-like disease progression and further control for neurodevelopmental or regulatory impacts of IL-6 deficiency. Nevertheless, as we saw a specific benefit of IL-6 knockout on memory function and we did not observe structural changes between genotypes, we believe no neurodevelopmental deficiencies were associated with IL-6 deletion in MRl/lpr mice, but further work is needed to confirm this interpretation.

Our experiments represent fundamental evidence supporting the necessity of prolonged IL-6 exposure in learning and memory in NPSLE-like disease; however, our experiments are only important initial steps in supporting the causality of this cytokine in these features. Several future experiments could build upon our findings. While Sakic et al. demonstrated induction of anhedonia in MRL/mpj mice using systemic IL-6 delivery via an adenoviral vector [46, 47], intrathecal delivery of IL-6 could determine if CNS-specific elevation of this cytokine is sufficient to induce learning or memory deficits or both. Similarly, intrathecal administration of anti-IL-6 or anti-IL-6-receptor blocking antibodies in MRL/lpr mice could further validate the CNS effects observed in our study.

Additionally, the response to treatment and the disease temporality of IL-6’s elevation in the brain still need to be characterized in the MRL/lpr model. Studying IL-6 expression and protein levels in the brain before development of NPSLE-like disease would allow us to better understand the early pathogenic role played by IL-6. The IL-6 knockout model studied in our experiments could be assessed periodically over time (e.g. 6 weeks, 10 weeks, 20 weeks) to identify when the memory improvement becomes apparent. Alternatively, intrathecal antibodies could be administered to inhibit IL-6 signaling at multiple time points in MRL/lpr mice to reveal when IL-6 exerts its potentially pathogenic effects. While such experiments represent possible future directions in firming the causality of IL-6 in NPSLE-like disease, the behavioral findings in IL-6 KO lupus mice from the present study represent valuable first steps in dissecting this potential etiology of NPSLE.

### Astrocytosis within the entorhinal cortex is associated with IL-6

Seeking a mechanism that may explain the behavioral findings, we assessed gliosis, or the pathologic activation and proliferation of microglia or astrocytes in CNS tissue [39]. We first quantified bulk expression of inflammatory genes in the cortex and hippocampus, two key brain regions critical in cognition, learning, and memory [101]. As many cortical regions could be involved, we chose to quantify gene expression in the entire cortex in these initial analyses. Should any bulk changes be found, our strategy was to identify the culprit region histologically.

Microglial *aif1* expression was increased in the hippocampus of IL-6 KO mice, initially leading us to consider that IL-6’s presence might paradoxically suppress microgliosis in this region. However, no corresponding increases in density of Iba1 + cells were found in the dentate gyrus. We chose the dentate gyrus a priori due to recent evidence implicating microglial pathology and reduction of dendrite density in this region in MRL/lpr mice [41]. Neither microgliosis nor structural pathology (i.e., abnormal layer thickness) were found in the CA1 region, chosen due to its known role in object-based memory task performance [83].

At this time, we cannot exclude the contribution of other detrimental mechanisms in the hippocampus (i.e., reduced neurogenesis) to behavioral findings of our study. Nevertheless, given the well-known direct effect of IL-6 on glial cells, we chose to prioritize this line of investigation. The amygdala exhibited no changes in glial density with IL-6 KO, which was expected due to the absence of differences in depression-like or anxiety-like behavior.

Expression of both microglial and astrocytic genes were found to be significantly decreased in the IL-6 KO cortex. However, none of the regions studied exhibited a decrease in microglia. We possibly did not capture the primary region contributing to our gene expression findings. Alternatively, IL-6 could play a more prominent role in the activation of astrocytes than microglia in MRL/lpr mice, and compensatory signals, such as TNF-α [38], sustain microglial activation in the absence of IL-6. Microglia-secreted IL-6 might be required to induce reactive astrocytes. Future experiments, such as cell-specific IL-6 knockdown, can tease apart the specific cell types responding to and producing IL-6 in this model to build upon our demonstration of IL-6’s role in neuropsychiatric lupus.

Nonetheless, astrocytosis under IL-6’s regulation was detected in the entorhinal cortex of MRL/lpr mice. While we did not observe associated differences in apoptotic cell numbers in IL-6 KO mice, the density of TUNEL-positive cells in both genotypes was comparable to data previously published in MRL/lpr mice [88]. Further work is needed to characterize neuronal death in the IL-6 KO MRL/lpr mouse, perhaps using Fluoro Jade B staining to better capture neuron loss in this model [102]. Prior work has shown that experimental lesions to the entorhinal cortex decrease novelty preference on OP and OR tasks [83]. Therefore, the observed entorhinal astrocytosis in IL-6 WT mice could replicate this functional pathology to induce the OP and OR deficits we observed.

Notably, functional subdivisions of the entorhinal cortex exist [83, 103, 104]. The lateral and medial subdivisions appear to mediate contextual or spatial memory, respectively. However, other studies support synergistic activity of the regions in either function [105]. We did not delineate the medial and lateral subregions on histologic analysis at this time. Both regions appeared to be impacted by IL-6 as we observed differences in both recognition and position memory. Furthermore, the lack of clearly defined boundaries at the inferior pole of the cortex complicates subdivision analyses. The lateral entorhinal cortex potentially comprised much of the assessed area, but slides from mice with more caudal slices (only a few tenths of a millimeter) could have contained larger portions of medial entorhinal cortex. On balance, we concluded that our analyses reflect gliosis within both subdivisions.

Similar to our findings, astrocyte-driven pathology has been implicated in the entorhinal cortex of patients with Alzheimer’s disease [106]. Increased density of astrocytes in this region was found on post-mortem histology. In fact, entorhinal atrophy likely occurs early in this neurodegenerative condition [107]. While the pathogenesis and histopathology of Alzheimer’s disease and NPSLE are obviously quite divergent, drawing parallels between the two diseases allows us to place our novel findings in the context of a well-studied disease that impacts cognition, learning, and memory. If clinical studies corroborate our animal model findings, the IL-6-astrocyte-entorhinal axis could prove a valuable etiologic mechanism and therapeutic target in NPSLE.

### Potential impact for human disease

It is important to frame our results within the current understanding of NPSLE’s potential pathogenesis. While focal deficits secondary to cerebrovascular insults produce acute events such as seizures, diffuse insults that have yet to be fully elucidated result in declines in cognition, learning, and memory [4, 6]. Clinical studies point to CSF inflammatory content, glial activation, and neuropathology [10, 11, 40]. Therefore, one important contribution to diffuse NPSLE may be an IL-6-associated neuroinflammatory process in key brain regions, leading to cognitive and memory decline.

Our findings cannot yet concretely identify the specific mediators of the IL-6 effect in MRL/lpr mice, but they indicate that IL-6, likely through glial cell activation, could impact a brain region vital to memory function. Neurons of the entorhinal cortex mediate memory functions by communicating with the hippocampus via glutamatergic signaling [108]. As IL-6 can stimulate B-cell immunoglobulin production [22], a reduction in anti-NDMA receptor antibodies or antibody deposition, an inflammatory nidus [109], could alternatively explain the restoration of memory function in IL-6 KO MRL/lpr mice. However, we did not observe IL-6 KO-associated reductions in anti-dsDNA immunoglobulins, so this alternate explanation appears less likely. Similarly, IL-6 is a lymphopoietic cytokine and the infiltration of T-cells into the CNS has previously been observed in MRL/lpr mice and patients [14, 62, 68]. Deletion of IL-6 could reduce the effect of these inflammatory lymphocytes in the brain. While our results point to stimulation of astrocytes in the entorhinal cortex by IL-6, follow up investigations could characterize the necessity of IL-6 in these mechanisms or others to further our understanding of how IL-6 mediates the entorhinal astrocytosis in this mouse model of human NPSLE.

Taken together, IL-6 likely plays a pivotal role in NPSLE; a conclusion supported by convincing clinical evidence in conjunction with strong support from the preclinical studies by Sakic et al. [46, 47], Nikolopoulos et al. [64], and our mechanistic observations in the present study. Follow up studies must validate IL-6’s causality in behavioral deficits, but our findings have promising translational impact. Targeting IL-6 in NPSLE therapy could resolve gliosis to restore normal regional brain homeostasis and memory.

IL-6-targeted therapy has already been assessed in SLE, using the anti-IL-6 receptor blocking antibody tocilizumab previously tested in a clinical trial [110]. This trial, however, found inconsistent effects on systemic disease, and tocilizumab was not approved or widely adopted for the treatment of SLE. Importantly, patients with CNS manifestations were not included in the tocilizumab trial. Moreover, no trials specific to neuropsychiatric lupus have tested this monoclonal antibody, or any other IL-6-targeting therapy for that matter.

Prior to such trials, CNS bioavailability of systemically-administered therapies which inhibit IL-6 must be assessed. For example, monoclonal antibody delivery to the CNS can be limited by the brain barriers [111]. Intravenously delivered tocilizumab showed likely sub-therapeutic, although detectable, levels in the brain during pre-clinical testing [112]. However, the increased presence of intrathecal antibodies and potential breakdown of the blood–brain and blood-CSF barriers [7, 10, 62] indicate that higher tocilizumab concentrations are possible in the brains of NPSLE patients. Additionally, techniques which optimize monoclonal antibody delivery to the brain tissue are rapidly advancing [111].

Future pre-clinical investigations should evaluate tocilizumab’s ability to replicate in MRL/lpr IL-6 WT mice (or other NPSLE models) the improvements seen in the behavioral deficits of the MRL/lpr IL-6 KO strain. Should cognition and memory similarly improve in vivo, tocilizumab clinical trials specific to neuropsychiatric manifestations of lupus would become compelling, potentially leading to the first treatment approved specifically for patients with NPSLE.

Diagnosing NPSLE often proves challenging [6]. While MRI studies point to frequent neuropathology, findings like white matter hyperintensities and calcifications can be non-specific [10]. Pathology in the entorhinal cortex, however, may present a new strategy for diagnosing neuropsychiatric lupus. Volumetric assessment of entorhinal atrophy needs to be directly assessed in NPSLE patients. Additionally, metabolite diffusion studies could focus on the entorhinal cortex to evaluate glial reactivity there. Both require validation, but imaging the entorhinal cortex as a non-invasive biomarker of NPSLE is a potentially exciting implication of our studies.

## Conclusions

NPSLE patients exhibit signs of neuroinflammation, and IL-6 appears associated with this pathology. Pre-clinical studies previously implicated acute IL-6 exposure in inducing anhedonia. However, whether chronic IL-6 exposure is required for the development of neuropsychiatric features in mice modeling NPSLE-like disease, specifically learning and memory deficits, was not previously known. Additionally, prior to our study, the degree to which the CSF composition of MRL/lpr mice overlapped with patient findings was unknown. We observed that the inflammatory content of MRL/lpr CSF resembles that of NPSLE patients. Specifically, IL-6 was elevated in the CSF, and we discovered positive associations between IL-6 serum concentrations and learning and memory deficits in MRL/lpr mice.

Studying IL-6 KO MRL/lpr mice, we uncovered evidence that IL-6 is responsible for defective learning and memory performance. Furthermore, constitutive IL-6 deficiency was associated with reduced astrocytosis in the entorhinal cortex, likely contributing to the improvement in neurobehavioral deficits observed in this strain compared to the IL-6 sufficient MRL/lpr strain. These findings support clinical imaging studies of the entorhinal cortex to determine if pathology in this region is an early indicator of NPSLE. Moreover, having only been assessed therapeutically for systemic manifestations, anti-IL-6 therapies, like tocilizumab, could have previously undiscovered therapeutic benefits specifically in NPSLE. Our findings potentially motivate the assessment of IL-6 targeting therapies in NPSLE-specific clinical trials.

### Supplementary Information

**Additional file 1:** Data and tables describing the results of MRL/lpr vs MRL/mpj behavioral testing (**Data S1**); analysis of microglial density in the hippocampus and its morphology in IL-6 KO vs IL-6 WT mice (**Data S2**); IL-6 cohort characteristics (**Table S1**); behavioral paradigms used (**Table S2**); glial gene expression in the brain of IL-6 KO vs IL-6 WT mice (**Table S3**).

## Data Availability

Supporting data is provided in the tables of this manuscript. Additional data is available upon reasonable request.

## Abbreviations

CNS: Central nervous system
CSF: Cerebrospinal fluid
FC: Fold change
GFAP: Glial fibrillary acidic protein
Iba1: Ionized calcium binding adaptor molecule 1
IL-6: Interleukin-6
KO: Knockout
SLE: Systemic lupus erythematosus
NPSLE: Neuropsychiatric SLE
WT: Wildtype
