# Supplementary material for: Constitutive knockout of interleukin-6 ameliorates memory deficits and entorhinal astrocytosis in the MRL/lpr mouse model of neuropsychiatric lupus
Source: J Neuroinflammation. 2024 Apr 10;21:89. doi: 10.1186/s12974-024-03085-9 (PMC11007930; doi:10.1186/s12974-024-03085-9)
Supplement: Supplementary file 1 — Additional file 1: Data and tables describing the results of MRL/lpr vs MRL/mpj behavioral testing (Data S1); analysis of microglial density in the hippocampus and its morphology in IL-6 KO vs IL-6 WT mice (Data S2); IL-6 cohort characteristics (Table S1); behavioral paradigms used (Table S2); glial gene expression in the brain of IL-6 KO vs IL-6 WT mice (Table S3). [file 12974_2024_3085_MOESM1_ESM.pdf]

Constitutive knockout of interleukin-6 ameliorates memory deficits and entorhinal astrocytosis in the MRL/lpr mouse model of neuropsychiatric lupus

Additional file

Data S1. MRL/lpr mice used in CSF analyses display expected NPSLE phenotype. Before carrying out proteomic analysis of MRL/lpr serum and CSF, we first wanted to confirm the expected NPSLE phenotype in this cohort of mice. We observed that the MRL/lpr mice intended for CSF analysis did display worse learning and memory, increased depression-like disease, and prominent anxiety-like behaviors. The lupus mice exhibited worse novelty preference on OP (MRL/lpr: 64% failed (n=39) vs MRL/mpj: 38% failed (n=39); *p=0.024), more immobility on Porsolt swim (MRL/lpr: 31.8±1.5% (n=38) vs MRL/mpj: 23.2±1.4% (n=39), ****p<0.0001), and reduced center time in the open field (MRL/lpr: 11.8+0.9% (n=39) vs MRL/mpj: 16.3±1.4% (n=40); **p=0.01). Respectively, these scores represent poorer learning and memory, the presence of depressive-like behavior, and increased anxiety-like behavior. Behavioral spectrometry did not reveal any difference between genotypes in baseline activity or locomotion.

Data S2. Microglial density and thickness of the CA1 region of the hippocampus in IL-6 KO and IL-6 WT mice. To further assess the hippocampal enrichment of the *iba1* gene in the hippocampus of IL-6 KO MRL/lpr mice, we quantified Iba1+ cells in the CA1 region of the hippocampus. Density of Iba1+ cells in CA1 was comparable between IL-6 KO and IL-6 WT MRL/lpr mice (IL-6 KO: 45.0±3.3 cells/mm^2 (n=8) vs IL-6 WT: 45.8±3.1 cells/mm^2 (n=8); p=0.959). Beyond gliosis, reductions in CA1 thickness can accompany learning and memory deficits. To assess this alternate hypothesis of our behavioral findings, we measured the ventral-dorsal thickness of the entire CA1 subregion and the CA1 neuronal soma layer. Neither the subregion (IL-6 KO: 807.5±18.8 µm (n=8) vs IL-6 WT: 830.0±27.2 µm (n=8); p=0.328) nor the neuron layer (IL-6 KO: 59.6±1.8 µm (n=8) vs IL-6 WT: 69.8±7.0 µm (n=8); p=0.574).

Condition	Genotype	Sex	Cohort	n	Terminal age (weeks)	Animal weight (g)
IL-6 WT	IL-6 +/+ MRL/lpr	Female	A + B	15	17.8 +/- 0.4	36 +/- 1
IL-6 KO	IL-6 -/- MRL/lpr	Female	A + B	15	17.2 +/- 0.3	33.6 +/- 0.9
p-value	-	-		-	0.276	0.078

Table S1. IL-6 cohort characteristics. Descriptive features and systemic disease assessment of the combined cohorts of IL-6 WT and KO mice. Within-genotype outcomes were statistically equivalent between cohort A and B. Means +/- standard errors are provided for each outcome measured. OD, relative optical density on ELISA. *p<0.05, **p<0.01

	Behavioral test	Abnormal murine behavior	Purpose / Human correlate
1	Behavioral spectrometry	-	Baseline locomotor activity
2	Open field task	Center avoidance	Anxiety
3	Object placement (OP)	Loss of novelty preference	Cognitive & position memory deficits
4	Object recognition (OR)	Loss of novelty preference	Cognitive & identity memory deficits
5	Porsolt swim (PS)	Increased floating (Learned helplessness)	Depression
6	Social preference (SP)	Social avoidance	Anhedonia, mood dysregulation
7	Elevated plus maze (EPM)	Open arm avoidance	Anxiety

Table S2. Behavioral tests used in assessment of MRL/lpr NPSLE-like disease.

Condition	Genotype	n	whole cortex expression: ***aif1***	whole cortex expression: ***gfap***	whole cortex expression: ***nos2***
IL-6 WT	IL-6 +/+ MRL/lpr	6	0.84 +/- 0.09	1.49 +/- 0.16	2.65 +/- 0.41
IL-6 KO	IL-6 -/- MRL/lpr	7	0.59 +/- 0.05	1.03 +/- 0.11	1.82 +/- 0.22
p-value	-	-	**0.049***	**0.044***	0.118
**Condition**	**Genotype**	**n**	**hippocampus expression: *aif1***	**hippocampus expression: *gfap***	**hippocampus expression: *nos2***
IL-6 WT	IL-6 +/+ MRL/lpr	5 - 6	0.47 +/- 0.02	2.04 +/- 0.18	1.25 +/- 0.22
IL-6 KO	IL-6 -/- MRL/lpr	6	0.62 +/- 0.05	1.92 +/- 0.14	1.58 +/- 0.33
p-value	-	-	**0.040***	0.613	0.435

~~Table S3. Glial~~ gene expression results in the brain. Results from qPCR analysis of cortical and hippocampal expression of key genes are provided. Cohort A was used for gene expression analyses, while Cohort B was reserved for histologic analysis. Mean fold change +/- standard error is provided for each outcome measured. Within-genotype outcomes were statistically equivalent between cohort A and B. *p<0.05.
